# CD19 Chimeric Antigen Receptor (CAR) T-Cell Therapy for Systemic Autoimmune Diseases

**DOI:** 10.7759/cureus.60924

**Published:** 2024-05-23

**Authors:** Ananya Reddy Cingireddy, Brianna Flores, John Wuthrich, Anirudh Reddy Cingireddy

**Affiliations:** 1 Internal Medicine, Mountain View Regional Medical Center, Las Cruces, USA; 2 Computer Science, East Central University, Ada, USA

**Keywords:** car t-cell therapy, safety, efficacy, systemic autoimmune diseases, cd19

## Abstract

A small number of drugs have been the sole stay of conventional treatment for autoimmune illnesses for the past 10 years. These medications have a number of side effects that restrict their usage and necessitate continuous administration to keep a patient in a state of remission. While many new treatments are being researched to address this problem, chimeric antigen receptor (CAR) T-cell therapy currently shows the greatest potential. Current medical guidelines do not currently advocate the use of this medicine because it is still in its early stages of development due to continuing clinical research. Therefore, the aim of this systematic review was to determine what new findings have been reported in recent studies about the safety and efficacy of CAR T-cells. From the nine studies collected in total, it was found that systemic lupus erythematosus (SLE) was the most often researched autoimmune disease. The CAR T-cell therapy had noticeable results after one to two months on average. The most frequent adverse effect was cytokine release syndrome (CRS), which was treated cautiously and infrequently necessitated extensive intervention. All serological tests showed improvement, and clinical remission was always achieved. This review concludes that, due to the one-time infusion and low adverse reaction rate, the therapy not only outperforms conventional drugs but is also more practical. There is even more cause to look forward to the full deployment of this innovative therapeutic alternative, as variations of the therapy are currently being explored.

## Introduction and background

By definition, systemic autoimmune diseases are entirely the result of autoantibodies produced due to the immunogenicity of a structure that can be either a cell, tissue, or antigen, resulting in active inflammation and damage of said structure, manifesting in various symptoms, such as but not limited to pain, limitation of movement, and dysfunction of an organ, ultimately leading to an overall reduced quality of life [1,2]. The various pathologies that are classified under systemic autoimmune diseases include systemic lupus erythematosus (SLE), systemic sclerosis, rheumatoid arthritis, Sjogren, myasthenia gravis, and even diabetes type I, amongst many others [3]. Currently, the only form of management is the control of symptoms via the use of corticosteroids, immunosuppressants, and biologics. As of this writing, there are no medically approved therapeutic options that are curative [4]. Adding to the plight is that the use of these medications is hindered by the high frequency and severity of side effects produced.

Owing to this dearth of effective options, novel strategies are actively being studied to address most forms of autoimmune disease, at least those that are common and globally prevalent. These include the use of allogeneic hematopoietic cell transplantation (HCT) that aims to replace the defective immune system of the patient or cell therapies that target autoreactive immune cells such as B cells and T cells [5]. Specifically, the latter involves the depletion of B cells that are primarily responsible for initiating autoimmunity [6]. This is where the use of chimeric antigen receptor (CAR) T-cell therapy comes in [7]. CAR T-cells are T cells that have undergone genetic modification, so they can produce antibodies specifically directed against CD19, which is a transmembrane protein found on B cells or even other antigens.

While originally used in experiments that assessed its capability to destroy or inhibit cancer cells, CAR T-cells hold much potential to address rheumatological illnesses, at least as far as producing and maintaining a prolonged remission is concerned. It should be noted that there are two further subtypes of CAR: chimeric autoantibody receptor T cell and CAR regulatory T cell (CAR treg) [8]. The former is a more tailored form of CAR used exclusively for autoimmune diseases while eschewing the side effects of CAR. However, the purpose of this systematic review is to search current literature on the effects of the original CAR T-cell therapy on autoimmune diseases only.

Research question

Firstly, it is important to know how efficacious is CAR T-cell therapy as compared to conventional treatment for autoimmune diseases. If so, the follow-up question is whether CAR T-cell therapy is more feasible than conventional therapy. Additionally, we need to consider if the positive outcomes outweigh the side effects brought about by CAR T-cell therapy. While we expect that not every autoimmune disease will be covered in literature, we still seek to determine how many of these autoimmune diseases have been experimented with CAR T-cell therapy and how effective the treatment was for each one. Lastly, it is important to determine what are the limitations in the literature regarding the findings of CAR T-cell therapy for autoimmune diseases.

Research objectives

Our primary objective is to find and compile studies published in the last five years that look at the use of CAR T-cell therapy for autoimmune diseases. Those studies will then be analyzed to see what recommendations can be generated regarding the implementation of CAR T-cell therapy for autoimmune diseases.

Research rationale

Conventional treatment for autoimmune diseases is limited to a few medications that have been depended upon entirely in the last decade. These drugs do not always bring about the required symptomatic relief in every patient, impart a host of side effects that limit their use, and require persistent use to maintain a state of remission. Numerous novel therapies are being studied to rectify this issue, but so far, CAR T-cell therapy holds the most promise currently. Due to ongoing clinical studies, the treatment is still in its infancy; thus, current medical guidelines do not recommend the use of this therapy yet. This is why it is pertinent to determine what recent studies have found regarding efficacy and safety so as to influence and inform clinical decisions regarding the prospective management of autoimmune diseases as a whole.

## Review

Methods

Searches were done primarily in Medline/PubMed database and use of the Google Scholar search engine. Only studies that were published in the last five years were considered, in order to keep reporting the latest findings only. The words used for the search were a combination of keywords related to CAR T-cell, CD19, and systemic autoimmune disease, amongst others in the form of Medical Subject Heading (MESH) terms or words within the title/abstracts (Table 1). The Population, Intervention, Comparison, Outcome (PICO) protocol was used to tailor the search, and all steps followed the Preferred Reporting Items for Systematic Reviews and Meta-Analyses (PRISMA) guidelines. The output of the search result was entirely filtered and scanned according to the inclusion criteria established. Eligible studies were then reviewed and mined for reportable data items. Most studies were locked behind paid access; thus, for some studies, only the abstract was used to populate the synthesis tables in this review.

**Table 1:** Search strategy terms

Search No	Search Terms
#1	CD-19 OR CD19 [Title/Abstract]
#2	CAR-T-CELL THERAPY [Title/Abstract]
#3	Autoimmune disease [MeSH Terms]
#4	Systemic autoimmune disease [Title/Abstract]
#5	Treatment efficacy [Title/Abstract]
#6	Treatment safety [Title/Abstract]
#7	#1 AND #2
#8	#1 AND #2 AND #3
#9	#1 AND #2 AND #4
#10	#2 AND #5
#11	#2 AND #6

**Figure 1:**
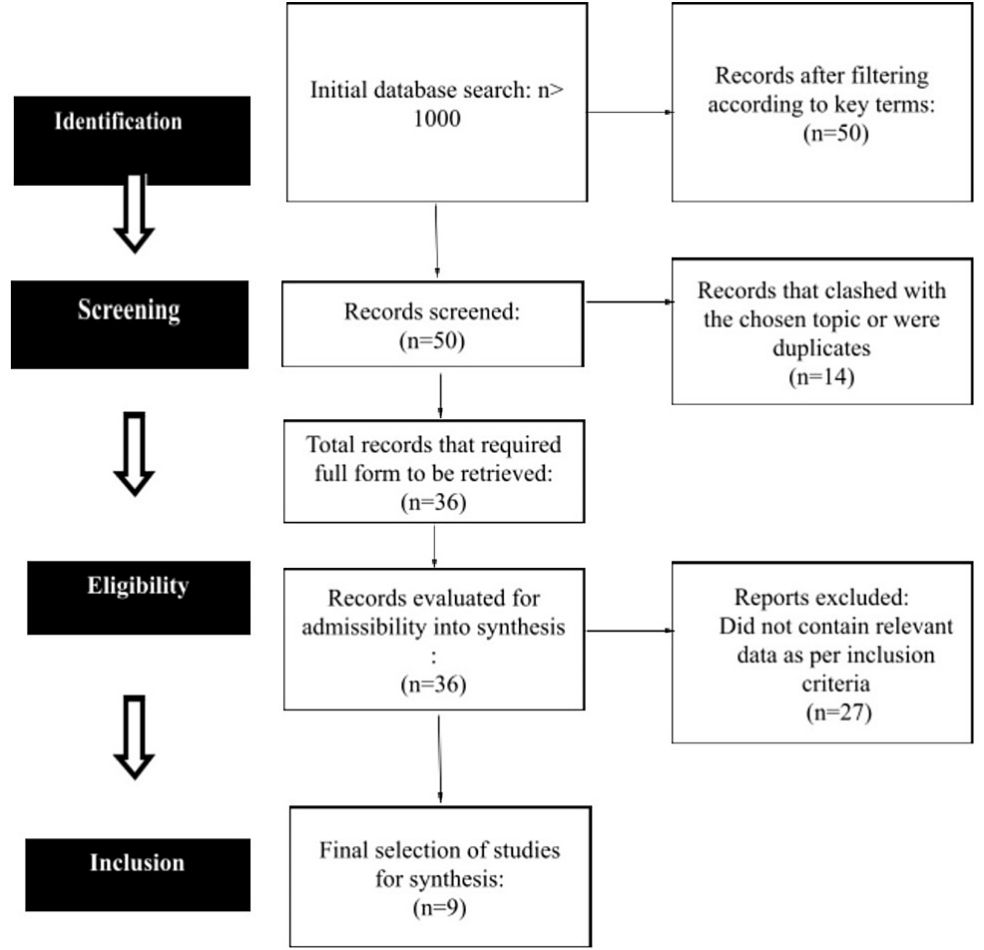
PRISMA flowchart showing the search strategy PRISMA: Preferred Reporting Items for Systematic Reviews and Meta-Analyses

Furthermore, the reference lists of collected articles and reviews were manually checked for additional studies that might have been overlooked during the initial database search in order to guarantee a thorough search. Additionally, for trials pertaining to CD19 CAR T-cell therapy in systemic autoimmune diseases, only completed trials were searched through clinical trial registries such as ClinicalTrials.gov; the purpose of this extra search step was to find unpublished or recently finished studies that might contain important data for this systematic review. The search strategy is shown in Table 2, and the final number of studies considered for inclusion is depicted in Figure 1.

**Table 2:** Search conducted on PubMed/Medline databases

Search number	Query	Filters	Search Details	Results
1	(CD-19[Title/Abstract]) OR (CD19[Title/Abstract])	From 2019 to 2024	("CD-19"[Title/Abstract] OR "CD19"[Title/Abstract]) AND (2019:2024[pdat])	5,481
2	CAR T CELL THERAPY[Title/Abstract]	From 2019 to 2024	("car t cell therapy"[Title/Abstract]) AND (2019:2024[pdat])	4,068
3	autoimmune disease[MeSH Terms]	From 2019 to 2024	("autoimmune diseases"[MeSH Terms]) AND (2019:2024[pdat])	97,817
4	systemic autoimmune disease[Title/Abstract]	From 2019 to 2024	("systemic autoimmune disease"[Title/Abstract]) AND (2019:2024[pdat])	966
5	treatment efficacy[Title/Abstract]	From 2019 to 2024	("treatment efficacy"[Title/Abstract]) AND (2019:2024[pdat])	9,460
6	treatment safety[Title/Abstract]	from 2019 - 2024	("treatment safety"[Title/Abstract]) AND (2019:2024[pdat])	582
7	((CD-19[Title/Abstract]) OR (CD19[Title/Abstract]) AND (2019:2024[pdat])) AND (CAR T CELL THERAPY[Title/Abstract] AND (2019:2024[pdat]))		("CD-19"[Title/Abstract] OR "CD19"[Title/Abstract]) AND 2019/01/01:2024/12/31[Date - Publication] AND ("car t cell therapy"[Title/Abstract] AND 2019/01/01:2024/12/31[Date - Publication])	1,166
8	(((CD-19[Title/Abstract]) OR (CD19[Title/Abstract]) AND (2019:2024[pdat])) AND (CAR T CELL THERAPY[Title/Abstract] AND (2019:2024[pdat]))) AND (autoimmune disease[MeSH Terms] AND (2019:2024[pdat]))		("CD-19"[Title/Abstract] OR "CD19"[Title/Abstract]) AND 2019/01/01:2024/12/31[Date - Publication] AND ("car t cell therapy"[Title/Abstract] AND 2019/01/01:2024/12/31[Date - Publication]) AND ("autoimmune diseases"[MeSH Terms] AND 2019/01/01:2024/12/31[Date - Publication])	10
9	(((CD-19[Title/Abstract]) OR (CD19[Title/Abstract]) AND (2019:2024[pdat])) AND (CAR T CELL THERAPY[Title/Abstract] AND (2019:2024[pdat]))) AND (systemic autoimmune disease[Title/Abstract] AND (2019:2024[pdat]))		("CD-19"[Title/Abstract] OR "CD19"[Title/Abstract]) AND 2019/01/01:2024/12/31[Date - Publication] AND ("car t cell therapy"[Title/Abstract] AND 2019/01/01:2024/12/31[Date - Publication]) AND ("systemic autoimmune disease"[Title/Abstract] AND 2019/01/01:2024/12/31[Date - Publication])	0
10	(CAR T CELL THERAPY[Title/Abstract] AND (2019:2024[pdat])) AND (treatment efficacy[Title/Abstract] AND (2019:2024[pdat]))		"car t cell therapy"[Title/Abstract] AND 2019/01/01:2024/12/31[Date - Publication] AND ("treatment efficacy"[Title/Abstract] AND 2019/01/01:2024/12/31[Date - Publication])	36
11	(CAR T CELL THERAPY[Title/Abstract] AND (2019:2024[pdat])) AND (treatment safety[Title/Abstract] AND (2019:2024[pdat]))		"car t cell therapy"[Title/Abstract] AND 2019/01/01:2024/12/31[Date - Publication] AND ("treatment safety"[Title/Abstract] AND 2019/01/01:2024/12/31[Date - Publication])	2

Eligibility Criteria

Inclusion criteria: The study included human studies that cover CAR T-cell therapy on patients with any form of autoimmune disease, regardless of age, gender, or ethnicity.

Exclusion criteria: Any in-vitro or in-vivo (non-human) studies done were not considered even if they held findings on the efficacy or safety of CAR T-cell. On the other hand, any human studies that did not report findings on treatment efficacy and safety or reported data on variations of CAR T-cell (which is not our objective) were also not considered. Lastly, all studies must focus on adult patients.

Data Extraction and Data Items

Data were extracted and recorded within Microsoft Excel software and included the following data items: author, study design, sample size, gender ratio, mean age, autoimmune disease diagnosed, assessment, treatment efficacy, side effects, and outcome.

PICO Framework

The PICO framework guided my research question and search strategy. The population includes patients with autoimmune disease of any type. The intervention is CAR T-cell therapy. The comparison is conventional medication, while the outcome is a reduction in the signs/symptoms.

Results

A total of nine studies were included in the final synthesis (Figure 1). With the exception of one, all of these were either case reports or case series. The most common autoimmune disease studied was SLE. Most patients were female and in their middle ages. Every treatment started with lymphodepletion and tapering of previous medications. In all these patients, symptomatic relief through conventional medications was not optimal. On an average of one to two months, the effects of the CAR T-cell infusion were significant. The most common side effect was cytokine release syndrome (CRS), which was managed conservatively and rarely required heavy intervention (Table 3).

**Table 3:** Synthesis of studies reporting on the safety and efficacy of CAR T-cell therapy in autoimmune diseases ACR-EULAR: American College of Rheumatology-European League against Rheumatism major clinical response; CRS: Cytokine release syndrome; DLBCL: Diffuse large B-cell lymphoma; DORIS: Definition of remission in SLE; EUSTAR: European Scleroderma Trials and Research Group activity index; EULAR: European Scleroderma Trials and Research Group activity index; HR: Hazard ratio; ICANS: Immune effector cell-associated neurotoxicity syndrome; LEMS: Lambert-Eaton myasthenic syndrome; SELENA: Safety of Estrogens in Lupus National Assessment; SSC-ILD: Systemic sclerosis-associated interstitial lung disease; SSDA: Sjogren syndrome disease activity index; MG: Myasthenia gravis; NHL: Non-Hodgkin lymphoma; NSIP: non-specific interstitial pneumonia

Author	Design	Sample Size	Gender Ratio	Mean Age	Autoimmune disease	Treatment type	Assessment	Treatment efficacy	Side effects	Outcome
Muller et al., 2024 [9]	Case series	15	-	-	SLE (8), idiopathic inflammatory myositis (3), systemic sclerosis (4),	Single infusion of CD19 CAR T-cells follow-up to 2 years	DORIS ACR-EULAR EUSTAR	SLE patients showed DORIS remission. All idiopathic inflammatory myositis patients showed an ACR–EULAR major clinical response. All systemic sclerosis patients showed a decrease in EUSTAR activity index.	Grade 1 CRS = 10 patients, Grade 2 CRS + immune effector cell-associated neurotoxicity + pneumonia = All patients	CD19 CAR-T-Cell was efficacious and safe in all studied autoimmune diseases.
Mougiakakos et al., 2021 [10]	Case report	1	1 Female	20 years	SLE	Single infusion of CD19 CAR-T-cells	Serological tests, clinical remission SELENA	dsDNA autoantibodies decreased from U/ml to 4 U/mL within 5 weeks. C3 and C4 levels increased to normal. Proteinuria decreased from 2000 mg to 250mg protein per gram of creatinine. SELENA decreased from 16 to 0.	None	CD19 CAR-T-Cell therapy is able to cause remission in SLE refractory to medication.
Mackensen et al., 2022 [11]	Case series	5	1:4	22 (6) years	SLE	After lymphodepletion, infusion of CAR T-cells	Serological tests Clinical remission DOEIS SLE disease activity index	Depletion of B-cells, normalization of anti dsDNA. DORIS criteria achieved in 5 patients after 3 months. SLE disease activity index lowered to 0 from 16 (8).	Mild cytokine release syndrome	Remission sans medication was maintained up to 8 (12) months. Reappearance of B-cells did not disrupt remission. Note: values presented as median (range).
Zhang et al., 2019 [12]	Case report	1	1 female	41 years	SLE + stage IV DLBCL	Following lymphodepletion, BCMA CD19 compound CAR T-cells were infused	Serological tests C3, C4 ANA Immunoglobins, plasma cell and leukocyte count PET CT	C3 and C4 levels normalized. All subtypes of ANA became undetectable. Immunoglobulin levels did not significantly decrease.	Not reported	CAR-T-Cells not only treated SLE but also treated DLBCL. Plasma cells were not detected in marrow four months after the initial infusion PET CT also showed absence of lesions.
Bergmann et al., 2023 [13]	Case report	1	1 male	60 years	Systemic sclerosis + Raynauds disease	Cessation of immunosuppression, followed by lymphodepletion, then infusion of CAR T-cells	Serological tests, clinical remission ANA Ig CT EUSTAR	Full b-cell depletion by 7th day. Restoration of CD4+ T cells on day 37 IgG levels did not drop below 700 mg/dL ANA reactivity fell to 0. Fibroblast activation decreased in myocardium (32.6%). Pulmonary fibrosis did not progress. Carpal arthritis improved after 3 months. Tender joint counts decreased from 22 to 3 EUSTAR activity was 0. Raynaud’s phenomenon attack frequency reduced. Skin fibrosis improved.	Cytokine release syndrome grade 1	CAR-T-Cell is effective and safe for systematic sclerosis.
Sheng et al., 2023 [14]	Case report	1	1 female	72 years	Sjogren disease + DLBCL	Fludarabine and cyclophosphamide administered prior to infusion of CAR T-cell	Serological tests, clinical remission ANA anti-rho-52 cytokine levels EULAR SSDA	Complete remission of Sjogren day 28. ANA and anti-ro-52 became negative. Cytokine levels returned to normal after half a year. EULAR SSDA went from 5 to 2.	Grade 2 cytokine release syndrome Grade neurotoxicity	CAR-T-Cells are effective in controlling symptoms of Sjogren.
Wang et al., 2023 [15]	Comparative study (propensity score matching study)	1363	-	-	B cell NHL vs B cell NHL + autoimmune disease	-	Serological tests, medication use	The group with autoimmune disease had a decrease of inflammatory markers, depletion of autoantibodies, and use of medications.	Both groups had similar incidence and severity of CRS and ICANS. Both groups had similar death and overall survival rates (HR=0.97 & 0.90 respectively)	CAR-T-Cell efficacy and safety is not impaired in patients with autoimmune disease (when treating B cell NHL).
Motte et al., 2024 [16]	Case series	2	1 female	33 years	MG +LEMS	CAR T-cell infusion	Acetylcholine receptor and voltage-gated calcium channel N-type autoantibody levels, B cell levels, QMG, MG-ADL, pulmonary function tests, examination of mobility	Resolution of trendelenberg sign, QMG score from 21 to 5, pulmonary vital capacity improved, mobility increased, QoL and MG-ADL improved.	CRS grade 2, mild arterial hypotension, mild neurological side effects	CAR-T-Cell therapy is efficacious in treating signs and symptoms of MG + LEMS.
			1 female	46 years				Independence from being wheel-chair bound, pulmonary vital capacity improved, mobility increased, QMG score from 21 to 5, QoL and MG-ADL improved.	CRS grade 1, local lymph node swelling	
Merckt et al., 2024 [17]	Case report	1	1 female	38 years	SSC-ILD + NSIP	Leukapheresis and lymphodepletion chemotherapy before infusion of CAR T-cells	Scl70, CRP, hsTNT and ANA levels, Modified Rodnan Skin Score, CT-scan, examination of motor function, Fcɣ-receptor-activating immune complexes	Scl70, CRP, hsTNT and ANA levels normalized. Skin fibrosis decreased, patient’s lung function improved, movement improved, immune complexes disappeared.	CRS grade 1	CAR therapy is effective and safe for treating Systemic sclerosis.

Discussion

This study is the first to examine the efficacy and safety of CAR T-cell therapy in treating autoimmune diseases. The majority of the studies that we found were either case reports or case series, and it was surprising that we could not locate any randomized control trials. A study design of this nature would have lent much more weight to the findings, especially given the small sample size in the case reports and series. Despite this, every case report demonstrated a robust methodology. While this review did not focus heavily on the formulation or the steps involved in creating CAR T-cells, we noted that, in all cases, the therapy was administered through a single infusion. This was sufficient to yield results, which adds merit to this therapy as a more feasible option for patients. Instead of having to take conventional medications on a regular and daily basis, patients only have to undergo one session of CAR T-cell therapy.

While all studies showed results, there was always an element of adverse reactions. However, these were limited to simple CRS. This is understandable, as most studies looking at clinical trials on treating hematological tumors report a high incidence of CRS, even culminating in severe grades [18]. However, in the context of using CAR T-cell therapy for autoimmune diseases, no test resulted in a CRS grade 3 or above. Other side effects were simply related to symptoms of headaches, tremors, and speech disturbances, which were mostly self-limiting. There were indeed instances of anemia and leukopenia, but these were most likely due to the initial lymphocyte depletion.

The effectiveness of CAR T-cell therapy largely depends on its ability to isolate target antigens and destroy them [19]. However, there can be instances where B cell populations, which were initially suppressed, rise up again post-treatment, rendering the CAR T-cells ineffective. This could ultimately result in a disease relapse. Although disease relapse has been reported in some studies [20,21], in the studies that we collected for this review, there was no incidence of relapse. However, it should be noted that, in certain cases, patient follow-up was discontinued after a certain period of time. It then stands to question how exactly long remission is maintained following the single infusion. A cohort study would have benefited this review, but in its absence, we are unable to fully assess the long-term effectiveness of treatment and the risk of relapse. Moreover, from what we have collected, all the included study's findings are, more or less, preliminary, but still important enough to merit planning long-term research to determine whether responses can be sustained for the necessary amount of time and whether the frequency and timing of CAR T-cell administration should be adjusted. In order to assess the place of CD19 CAR T-cell therapy in rheumatological disease management, this may require not only in-vitro or in-vivo models but human studies too; only then can the results be compared directly with currently available treatments as well as value-based analyses performed.

Furthermore, there may be instances where there is a poor response to CAR T-cell therapy, or in other words, resistance. This resistance to therapy has been observed in some cases, although the specific circumstances and reasons for this resistance need further investigation [22,23]. Perhaps more limelight should be on the several mechanisms of action of the therapy. The distinct and rapid elimination of B cells that create autoreactive B cell clones is one of the potential therapeutic mechanisms of CD19 CAR T-cell therapies for systemic autoimmune diseases that can be focused on as it is unclear exactly how these B cells are being lost. Current theory discusses how it is thought to set off the immune system's reset to a tolerogenic state, which allows B cells to regenerate their repertoire. 

The use of CAR T-cell therapy, originally developed for treating tumors and now being applied to autoimmune diseases, represents a significant innovation [24]. It is important to remember that a CAR T-cell is composed of four parts: the antigen-binding domain, hinge domain, transmembrane domain, and intracellular domain. These components form the fundamental structure of CAR T-cells, but there is room for improvement and variation. This is where CAAR T (chimeric autoantibody receptor T cells) come in [25]. Because the principle of CAR T-cell therapy lies in its ability to deplete B cells, this will always lead to adverse effects, since B cells are not inherently malicious, but an integral part of our immune system. It is only their unchecked behavior in autoimmune diseases that warrant their suppression; thus, to avoid any issues resulting from low levels of B cells, CAAR T cells, which have antigen-binding domains made of self-antigens, can potentially mitigate these effects.

In addition to CAR T-cells, there are other variations such as CAR-NK cells and CAR tregs, which have been explored in various research studies [26,27]. There is also potential for combining different therapies along with CAR T-cells to achieve a synergistic effect, enhancing the overall efficacy of management for rheumatic illnesses. This approach has been reported in several studies, showing exactly how much evolution this therapy is undergoing.

Limitations

Our systematic review faced both minor and major limitations in reporting the efficacy and safety of CAR T-cell therapy. Firstly, related to our end, we were unable to retrieve most articles that may have held crucial info. Secondly, related to the studies collected, since they were primarily case reports and case studies, the small sample sizes were too small for us to consider the findings applicable to larger populations. In other words, the generalizability of the findings was poor. Furthermore, only a few select autoimmune diseases are being focused on, leaving a vast number of other minor autoimmune diseases untouched.

A major limitation was regarding the therapy itself. One of the significant challenges is the initiation of humoral and cellular immune responses in patients due to one of the domains of the CAR T-cell build, which is not derived from humans in most commercial products. This can lead to the generation of anti-CAR antibodies, potentially clearing the therapeutic CAR T-cells and reducing the efficacy of CAR T-cells upon reinfusion. These immune reactions can affect CAR T-cell growth and continuity, potentially affecting the overall clinical response. Another concern is the life-threatening hyperinflammatory syndrome, secondary haemophagocytic lymphohistiocytosis (sHLH), or macrophage activation syndrome (MAS), which normally can occur in patients with severe autoimmune diseases but is also reported following CAR T-cell therapy. This must be differentiated CRS, but that in itself has not been successfully done. 

A second major limitation was the possibility of spillover effects of conventional medications that may have contributed to the improvement in the patient’s parameters. Additionally, since every infusion was preceded by leukodepletion, the effects of that on lowering B cells cannot be overlooked.

Lastly, the shift to a more specific CAAR T-cell therapy in recent studies could potentially render CAR T-cell therapy obsolete, as far as autoimmune diseases are concerned.

## Conclusions

This systematic review gives a snapshot of the most recent information on how effective and safe CAR T-cell therapy works as a treatment for systemic autoimmune diseases. The therapy does not only excel conventional medications but also is more feasible owing to the one-time infusion and minimal adverse reactions. The fact that variations of the therapy are also being studied adds further reason to anticipate the full implementation of this novel therapeutic option. We recommend that future endeavors in studying this form of management determine workarounds for the immunogenicity of CAR T-cells, the ability to distinguish CRS from MAS, and, if possible, prevent any adverse reaction at all. It is also important for larger randomized controlled trials to make a proper assessment to rule out confounding factors such as possible effects of conventional medications and preliminary leukodepletion before infusion of CAR T-cells. A comparative study between the various subtypes of CAR T-cells will also be needed eventually.
